# Opacification of refractive bifocal intraocular lens in one month: Three case reports

**DOI:** 10.1097/MD.0000000000028757

**Published:** 2022-02-04

**Authors:** Yanfeng Zeng, Min Liang, Cheng Fan, Sen Xu, Fengting Liu, Xiaoli Zhou, Xin Tan, Xiaoqin Wang

**Affiliations:** Li Xiang Eye Hospital Affiliated to Soochow University, Suzhou, Jiangsu Province, China.

**Keywords:** injector cartridge, IOL, LS-313 MF15/30, opacification

## Abstract

**Rationale:**

Multifocal intraocular lenses (IOLs) are used widely. However, the discovery of LS-313 MF15/30 (Oculentis B.V.) opacity during surgery has not yet been reported. This article reports 3 cases of LS-313 MF15/30 (Oculentis B.V.) IOL opacity found during cataract surgery implantation within 1 month.

**Patient concerns:**

Three patients underwent cataract surgery, and opacification of their IOL (LS-313 MF15/30, Oculentis B.V.) was found intraoperatively.

**Diagnosis:**

The patient was diagnosed with a postoperative intraocular opacity.

**Interventions:**

In case 1, the surgeon scrubbed the IOL with intraocular perfusion fluid and a gelatin sponge swab to reduce opacity in the central optical area of the IOL and then implanted it into the capsule bag. In case 2, the surgeon used the infusion-aspiration polishing mode for cleaning. To avoid IOL wear and bag damage, washing was stopped when turbidity in the center of the optical area was reduced. In case 3, we learned from our previous experience that the surgeon cut the IOL into 2 pieces and moved it out at the main incision, which was replaced and implanted with a brand new IOL, after the implanted IOL was again found cloudy.

**Outcomes:**

In case 1, more than 10 months after the surgery, the IOL was restored to transparency, no obvious eye discomfort was noted, and uncorrected visual acuity was 20/25. In case 2, the patient's IOL surrounding area was still partially turbid after more than 10 months of follow-up. In case 3, the patient's uncorrected visual acuity on postoperative day 1 was 20/20, and the best-corrected visual acuity was 20/20.

**Lesson:**

There are many reasons for the opacification of the IOL. In addition to the patient's own factors, the material, production, and packaging of the IOL, as well as the influence of external environmental temperature, the influence of the IOL implant instrument should not be ignored and needs to be considered.

## 1 Introduction

The opacity of intraocular lenses (IOLs) directly affects the visual acuity and quality of life of patients. The incidence of IOL opacity is low, and IOL replacement is often needed if the condition is serious. There are many reasons that may be related to IOL opacity, such as the materials, manufacturing process, packaging, and other factors. It may also be related to environmental temperature and surgical factors, such as surgical procedures, intraoperative IOL implant selection, and the patient's own factors, such as metabolic status or systemic disease.^[1]^

IOL opacifications such as hydrophobic, hydrophilic acrylate, polymethylmethacrylate, silicone, and other materials have been reported. Different types of materials are associated with different types of IOL opacifications.^[2]^ An analysis reported that the most common opacified lenses were caused by hydrophilic IOLs and the mean time of appearance of lens opacification was 14.93 ± 17.82 months.^[3]^ Some studies have found that opacification of hydrophobic IOLs has been reported in the literature as a late and permanent change, such as Akreos Adapt AO (Bausch & Lomb)^[4,5]^ and Hydroview (Bausch & Lomb).^[6]^ Opacified hydrophilic acrylic IOLs may also be associated with intraocular air or systemic disease.^[7]^ Cases in which clouding and opacification of the IOL occurred intraoperatively within a few seconds of implantation into the capsular bag after routine phacoemulsification because of temperature have also been reported.^[8–10]^ Furthermore, reversible opacification of hydrophobic acrylic IOLs has also been reported.^[11]^

As the number of patients choosing multifocal IOLs has gradually increased, many different types of multifocal IOLs have become available. LS-313 MF15/30 (Oculentis B.V.) provides patients with better visual acuity than monofocal IOLs. It is designed for folding prior to insertion and is manufactured from a medical-grade, low-water (25%) ultrahigh-purity hydrophilic material with hydrophobic surface properties. This article reports 3 opacifications of the LS-313 MF (Oculentis B.V.) 1 month before and after implantation of the IOL capsule during routine surgery. All surgeries were performed by the same surgeon who specialized in surgery.

## 2 Case presentation

### 2.1 Case 1

In November 2019, to meet the patient's expectations of high-quality postoperative vision, including distant and near vision, a 55-year-old man with a complicated cataract was scheduled to undergo left eye phacoemulsification assisted by the femtosecond laser technique with LS-313 MF30 (Oculentis B.V.) implantation. His pre-operative best-corrected visual acuity (BCVA) was counting fingers (FC)/20CM.

**Figure 1:**
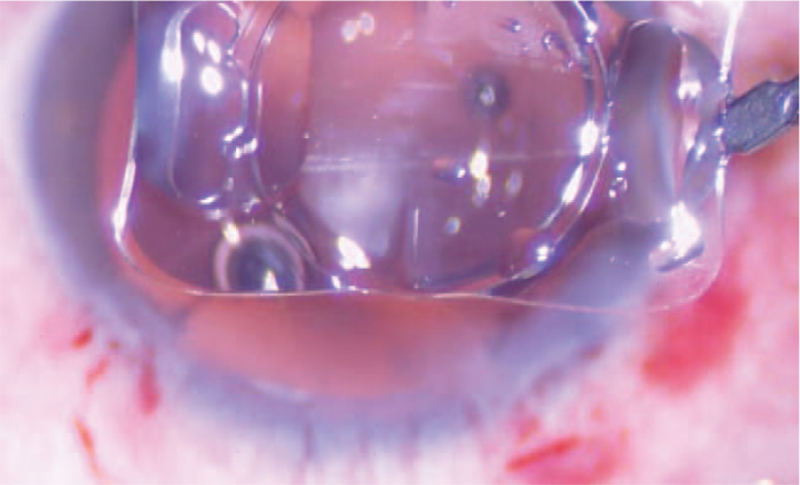
The IOL was opacification before implantation. IOL = intraocular lens.

**Figure 2:**
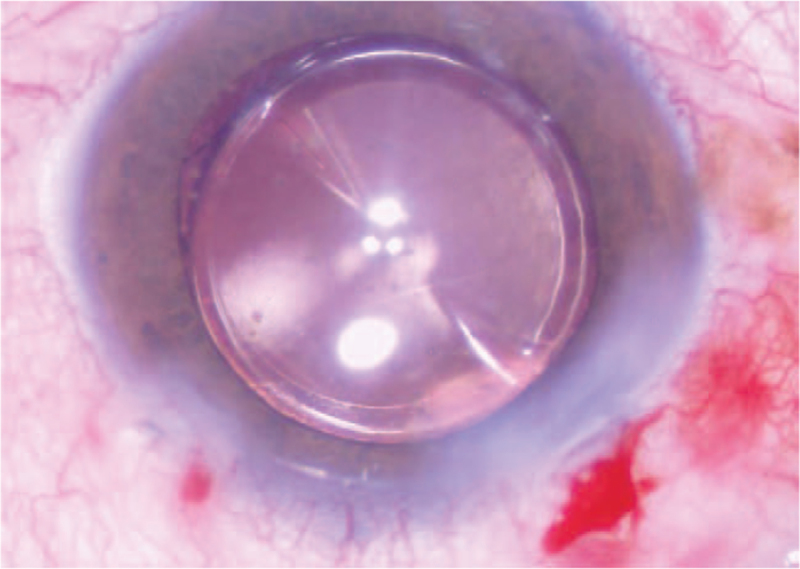
The opacity in the central optical area of the IOL lessened. IOL = intraocular lens.

**Figure 3:**
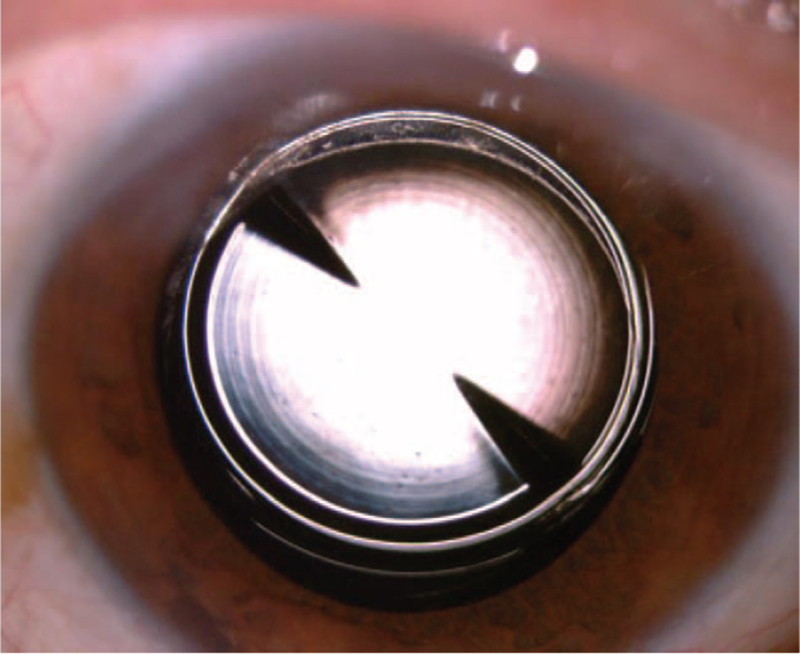
The patient's IOL was basically restored to transparency. IOL = intraocular lens.

The pupil was dilated with tobicamide 3 times pre-operatively. Promethaine was anesthetized 3 times prior to the operation. After using a femtosecond laser, the phacoemulsification was uneventful and was performed under topical anesthesia. The IOL was opacified before implantation (Fig. 1) immediately after the device nurse placed the IOLs on butterfly model cartridges (Viscoject Eco 2.2 VE2200, Medicel AG), which had been injected with viscoelastic substances. The surgeon scrubbed the IOL with intraocular perfusion fluid and gelatin sponge swab. The opacity in the central optical area of the IOL decreased and the IOL was implanted into the capsule bag (Fig. 2). The patient's IOL opacity did not change significantly on the first postoperative day. The postoperative uncorrected visual acuity 1 day after the operation was 20/40. Her BCVA was 20/40. The intraocular pressure was 14 mm Hg. The IOL opacity of the patient showed no significant change at 1 week and 1 month postoperatively; however, the uncorrected visual acuity was 20/25. More than 10 months after the surgery, the IOL was restored to transparency (Fig. 3), no obvious eye discomfort was noted, and uncorrected visual acuity remained at 20/25.

### 2.2 Case 2

**Figure 4:**
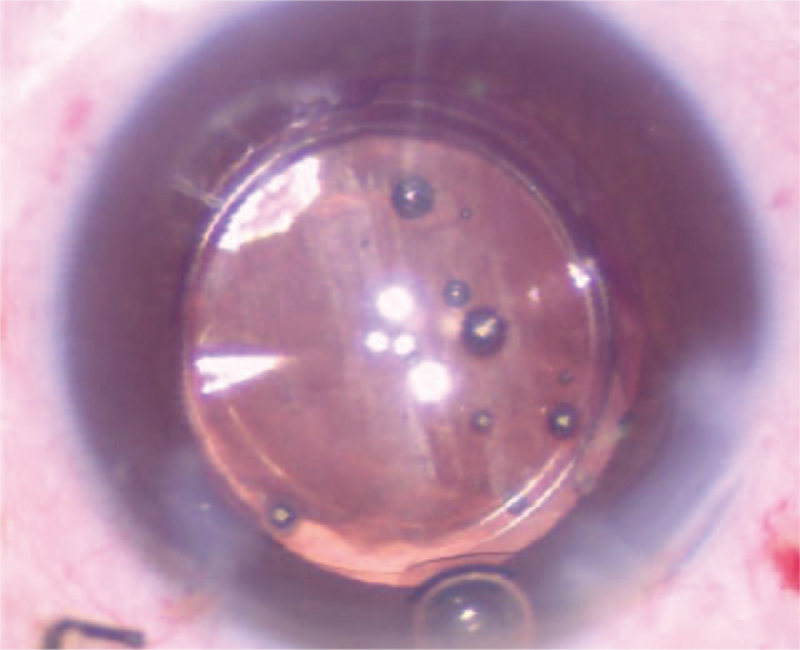
The optical region became cloudy, as soon as it was implanted into the eye.

In December 2019, a 70-year-old man with a cortex and nucleus cataract underwent right eye phacoemulsification with a +11.5D LS-313 MF15 (Oculentis B.V.) implantation. The patient's pre-operative BCVA was 20/1000. A perioperative regimen of mydriasis by tropicamide was routinely used 3 times every 10 minutes, and superficial anesthesia with proparacaine was administered 3 times before the operation. As soon as the optical region was implanted into the eye, it became cloudy (Fig. 4).

**Figure 5:**
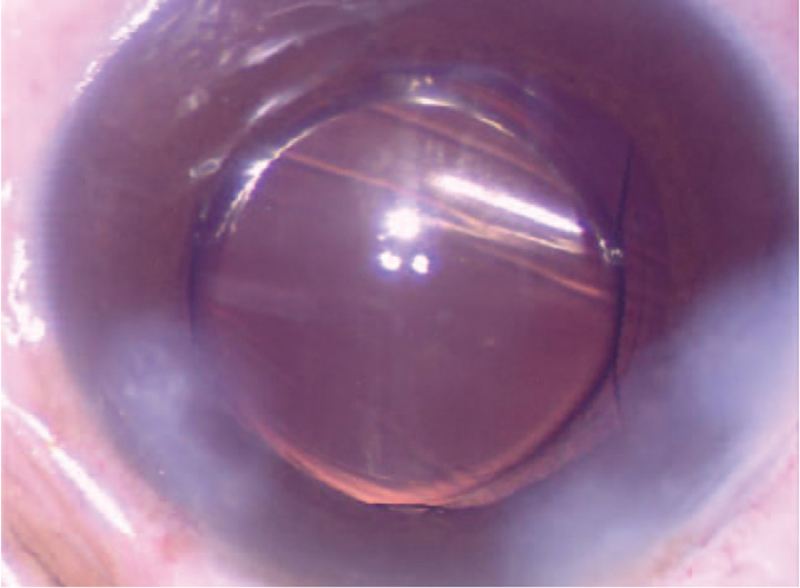
The washing was stopped when the turbidity in the center of the optical area was lessened.

**Figure 6:**
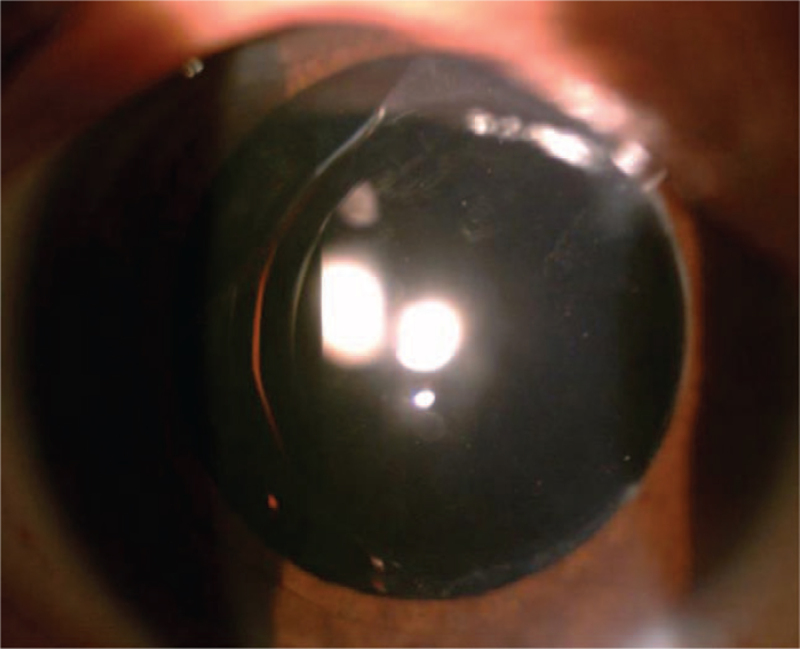
It was found that the patient's IOL surrounding area was still partially patellar turbidity after the operation. IOL = intraocular lens.

Thus, the infusion-aspiration (IA) polishing mode was used for cleaning. To avoid IOL wear and bag damage, washing was stopped when turbidity in the center of the optical area was reduced (Fig. 5). The IOL opacity in this patient showed no significant change at the follow-up 1 week and 1 month after surgery, and the uncorrected visual acuity was 20/20. It was found that the patient's IOL surrounding area was still partially turbid (Fig. 6) after the operation, and the patient came for a follow-up visit for more than 10 months.

### 2.3 Case 3

**Figure 7:**
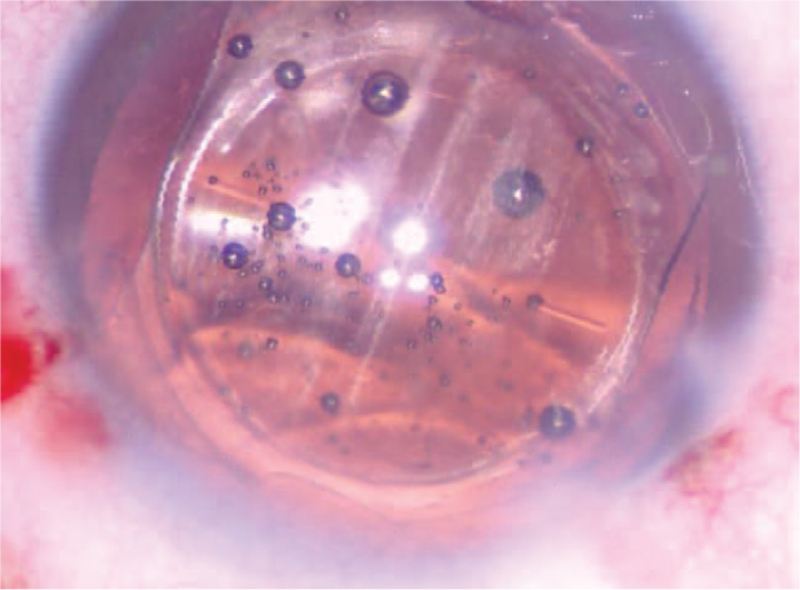
After IOL was implanted into the bag IOL was again found cloudy. IOL = intraocular lens.

**Figure 8:**
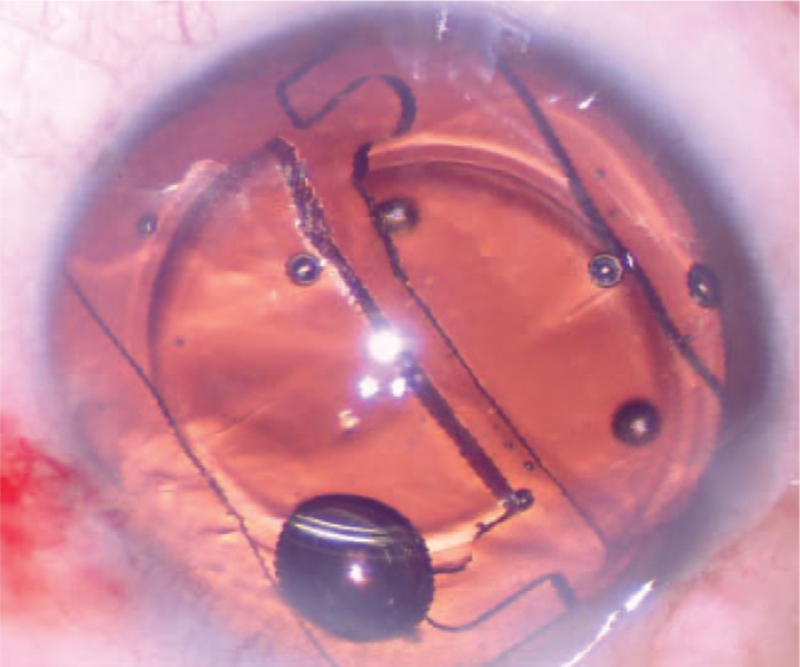
The IOL was cut in half and removed from the primary incision. IOL = intraocular lens.

**Figure 9:**
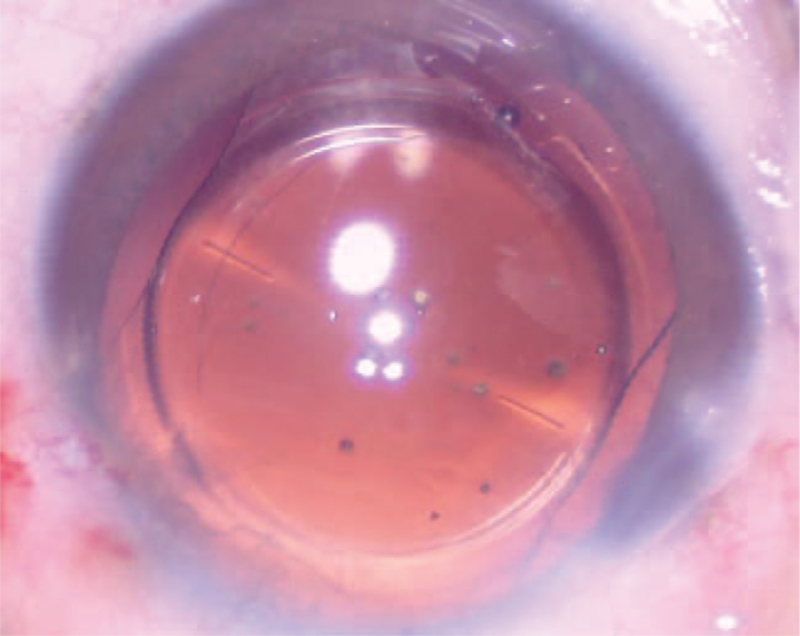
The IOL after replacement was transparent. IOL = intraocular lens.

In December 2019, a 71-year-old man with cortex and nucleus cataracts underwent left eye phacoemulsification with LS-313 MF30 (Oculentis B.V.) implantation. The patient's pre-operative BCVA was 20/300. The patient was treated with a perioperative regimen of mydriasis with tropicamide 3 times every 10 minutes and superficial anesthesia with proparacaine 3 times. After the IOL was implanted into the bag, it became cloudy (Fig. 7). The surgeon then cleaned the IOL using the infusion-aspiration polishing mode for approximately 10 minutes, but the opacity did not change significantly. Because the IOL opacity of the above 2 patients showed no significant changes at the follow-up 1 week and 1 month after surgery, the surgeon could not know what time it would clear; therefore, the IOL was cut into 2 pieces and moved out from the main incision (Fig. 8). After replacement, the IOL was transparent (Fig. 9). On postoperative day 1, his uncorrected visual acuity was 20/20, and his BCVA was 20/20. During our follow-up observations up to 6 months, the IOL remained transparent.

## 3 Discussion

With the continuous improvement in cataract patients’ requirements for visual quality, the variety of multifocal IOLs available for selection is gradually increasing. LS-313 MF15/30 (Oculentis B.V.) provides patients with both near- and far-vision. It is designed for folding prior to insertion and is manufactured from a medical-grade, low-water (25%), ultrahigh-purity hydrophilic material with hydrophobic surface properties. In contrast to previous reports described by Maria,^[12]^ Mariann et al.^[13]^ on pure hydrophilic acrylic IOLs, the opacifications of hydrophilic acrylic IOLs are mainly related to the deposition of calcium and phosphorus. In addition, unlike other IOL turbidity reports of similar materials,^[9]^ the IOL turbidity in these 3 cases was limited, and the stripes were dirty.

We contacted the Oculentis manufacturer after this situation had occurred. The problem originated from the injector cartridge according to the replies. Two types of injector cartridges are available on the market. The oldest butterfly model cartridges are made from polypropylene (PP), which contains a generic additive called glycerol monostearate (GMS) as a slip agent. Suppliers Medicel (only the Eco version), RET Korea, and many others. Injector cartridges were injection-molded from PP compounded with approximately 10% to 15% GMS as an additive. The GMS must migrate to the surface to form small islands of GMS; this effect is called blooming and provides PP with sliding properties. Without a GMS or to a small GMS, the IOL may be stuck in the injector and be damaged. This is also why the shelf life of this type of injector cartridge is limited to a few years as the blooming time becomes too long, resulting in more stripes. The PP GMS blend is unstable, and glycerol monostearate, commonly known as GMS, is monoglyceride. Its molecular formula is C21H42O4, which has 2 configurations: 1-Mg and 2-Mg. In addition, its molecular weight is 358.56. Generally, oily, fatty, or waxy, the color is light yellow or ivory with a grease taste, or tasteless. It is a stearic acid glycerol ester. GMS is an important biological plastic additive owing to its unique surface activity characteristics. In the plastics industry, it is an internal lubricant for polyethylene processing, which can promote smooth gelation and improve fluidity. It is widely used in the food and chemical industries.^[14]^ It is the most commonly used emulsifier in food processing.^[15]^ GMS is digested and decomposed into fatty acids and polyols in the human body, which can be absorbed or excreted by the human body. However, because the microenvironment in the human eye is different from that in other parts of the body, there is no literature that clearly indicates the metabolic situation in the eye.

As a result, we speculate that the opacification of the 3 IOLs with the same performance in this report is due to the injector cartridge microparticles coming out. During lens injection, powder-like particles can appear on the optical surface of the lens. In addition, the longer the contact time between the IOL and injector cartridge (Viscoject Eco 2.2VE2200, Medicel AG) during surgery, the less likely it is that the opacity will disappear. Moreover, due to the lack of reports on the opacity of IOL caused by GMS, the analysis of the long-term intraocular safety of GMS requires further follow-up studies.

To avoid the recurrence of this IOL opacity, we require the manufacturer to reconfigure another model of the IOL injection system (VISject Bio 1.8, Medicel AG), which uses engineered biofilms to offer outstanding gliding properties for IOLs to be safely injected through even smaller incision sizes while avoiding the transfer of additives to the lens surface. It is a biological material that exists naturally in the human eye. LS-313 MF15/30 (Oculentis B.V.) has not been cloudy until now.

## 4 Conclusion

The opacification of 3 cases of intraoperative IOL (LS-313 MF15/30, Oculentis B.V.) was caused by the release of GMS coating particles from the injector cartridge (Viscoject Eco 2.2VE2200, Medicel AG) to the IOL surface; however, the long-term intraocular safety of GMS was uncertain. Therefore, we recommend an IOL injection system (VISject BIO) containing an engineered biofilm coating for implantation of LS-313 MF15/30 (Oculentis B.V.) 1.8, Medicel AG) to avoid IOL opacification. There are many reasons for the opacification of the IOL in cataract surgery. In addition to the patient's own factors, the material, production, packaging of the IOL, and influence of external environmental temperature, the influence of the IOL implant instrument should not be ignored and should be considered.

## Acknowledgments

The authors would like to thank Li Xiang Eye Hospital affiliated with Soochow University and the Oculentis manufacturer. We also thank the patients for their support.

## Author contributions

**Funding acquisition:** Cheng Fan, Xiaoqin Wang.

**Methodology:** Xin Tan.

**Resources:** Min Liang, Xiaoli Zhou.

**Software:** Fengting Liu.

**Writing – review & editing:** Yanfeng Zeng, Sen Xu.

## Correction

Several author degrees have been corrected. Min Liang and Xin Tan have Masters of Medicine (MM) instead of the original MD. Sen Xu, Xiaoli Zhou, and Fengting Liu have Bachelors of Medicine (BM) instead of the originally listed MD.
