# Opacification of refractive bifocal intraocular lens in one month: Three case reports: Erratum

**DOI:** 10.1097/MD.0000000000028905

**Published:** 2022-02-18

In the article, “Opacification of refractive bifocal intraocular lens in one month: Three case reports”,^[1]^ which published in Volume 101, Issue 5 of *Medicine*, Dr. Cheng Fan's name appeared incorrectly as Licheng Fan. This has been corrected.
